# Impact of lifestyle modification on glycemic control and cognitive function among Type II diabetes mellitus patients

**DOI:** 10.2144/fsoa-2022-0060

**Published:** 2023-03-09

**Authors:** Iman I Salama, Samia M Sami, Somaia I Salama, Ghada A Abdel-Latif, Ahmed Aboulghate, Hala M Raslan, Amira Mohsen, Hanaa Rasmy, Mona Hamed Ibrahim, Mona MF Ganem, Aida M Abdelmohsen, Lobna A El-Etreby, Nihad A Ibrahim, Walaa A Fouad, Sherif E El-Deeb

**Affiliations:** 1 Community Medicine Research Department, National Research Center, Cairo, 12622, Egypt; 2 Child Health Department, National Research Center, Cairo, 12622, Egypt; 3 Internal Medicine Department, National Research Center, Cairo, 12622, Egypt; 4 Clinical & Chemical Pathology Department, Centre of Excellence, National Research Centre, Cairo, 12622, Egypt

**Keywords:** cognitive function, glycemic control, lifestyle modification, T2DM

## Abstract

**Aim:**

Assessing impact of lifestyle modification on Type 2 diabetes mellitus (T2DM) glycemic control and cognitive function.

**Subjects & methods:**

Prospective study was conducted on T2DM patients (92 patients as interventional group and 92 patients conventional therapy).

**Results:**

After 6 months, significant improvements of HbA1c, oxidant and antioxidant, lipid profile, and cognitive function among only the interventional group (p < 0.05). Using logistic analysis, conventional therapy, DM duration >10 years, lower education, HbA1c baseline >7 were significant predictive risks for uncontrolled DM (AOR 4.2, 2.9, 2.7 and 2.2, respectively). While, conventional therapy, baseline mild cognitive impairment (MCI) and females were significant risks for MCI (AOR 11.5, 10.8 and 4.8, respectively).

**Conclusion:**

Lifestyle modification is a very important for glycemic control and cognitive function.

**Clinical Trial Registration**: NCT04891887 (ClinicalTrials.gov)

## Plain language summary

The study aimed at assessing the effect of lifestyle modification program on Type 2 diabetes mellitus (T2DM) patients. The program contents include maintaining healthy diet depending on glycemic index and CHO counting, adjusting cholesterol level, regular physical activity for at least 30 minutes; 3–5 days per week, weight loss and maintaining an appropriate weight, controlling the blood pressure, smoking cessation and practicing mental activity. After 6 months, there was a significant improvement in glycemic control, cognitive function, oxidant and antioxidant and lipid profile levels among patients participating in the program but not among those remained on the conventional therapy.

## Graphical abstract

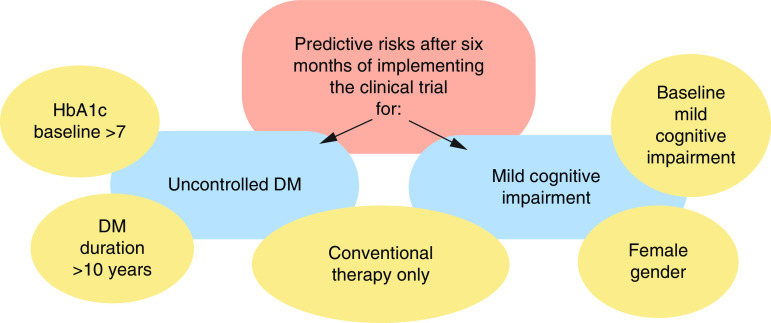

Diabetes mellitus (DM) is considered as one of the commonest non-communicable diseases worldwide and characterized by its chronicity and many severe complications. The global prevalence of diabetes is estimated to be 9.3% (463 million people) in 2019, rising to 10.2% (578 million) by 2030 and 10.9% (700 million) by 2045. The majority of DM patients are living in low-and middle-income countries [1]. Over 4 million people were estimated to die from diabetes-related causes in 2019 and in Egypt, the estimated national prevalence of type 2 DM (T2DM) among adults is 15.2% [2].

DM burden is mainly associated with obesity, unhealthy diet, and physical inactivity [3]. Diabetes education is considered an essential tool in its management. Dietary programs and physical activity interventions are essential in the treatment of DM and preventing its complications. Uncontrolled blood glucose levels among DM patients leads to many vascular and neuropathic complications which affect brain function. Therefore, these patients are more prone to cognitive decline [4,5]. The association of COVID 19 with type 2DM makes the whole condition at a higher risk of poor outcomes. For this reason, from now on, diabetes care has to include a careful, holistic evaluation of all the patients affected by COVID-19 [6]. Recently, new-onset Type 1 diabetes and ketoacidosis have increased worldwide during the pandemic period. Based on these findings, continuous and repeated educational diabetes awareness should be delivered to physicians, caregivers, and the public to improve health outcomes in the world and change trends DM [7]. A systematic review and meta-analysis documented that diet quality may affect disease severity in the recent pandemic period [8].

There were many methods used for DM education and lifestyle modifications such as individual face to face education and small group education [9]. Moreover, health information technology (via mobile, e-mail, and web sites) showed evidence in enhancing chronic disease management [10]. In Egypt, only 17.8% of T2DM patients achieved the targeted glycemic control according to the recommendations of the international guidelines [11]. The objective of the current study was to assess the impact of implementation of a six months lifestyle modification program on glycemic control and cognitive function among T2DM patients.

## Subjects & methodsf

The current study was a part of a project, which was carried out during the period from Feb 2016 till June 2019. It was a prospective clinical trial. Participating in it was completely voluntary. Participants were free to withdraw from the trial at any time and for any reason. After having the ethical approval for conducting this project, participants' enrolment was carried out before registering the project in ClinicalTrial.gov, as this registration was not requested from the ethical committee or the funding agent.

### Baseline assessment

It was a cross sectional study, using a convenience sampling, carried out on 184 consecutive T2DM patients attending outpatient clinics of the Medical Research Center of Excellence of the National Research Center (NRC). Inclusion of patients depends mainly on their acceptance to participate, at ease to be followed up and assessed due to their work place proximity. It is a type of non-random sampling. The participants' recruitment was carried out during the period from 15th August 2016 till 14th July 2017. Patients were 76 males and 108 females; aged from 40 to 65 years. All patients were literate and were able to complete the tests of cognitive function. The exclusion criteria were T2DM patients with history of head trauma, stroke, transient ischemic attack, brain tumor, epilepsy, psychiatric disease, cardiac or liver failure, and visual or hearing disabilities.

#### A face-to-face interview

was carried out with all participants to collect data about demographic data, medical history and assessment of cognitive function. Data included age, gender, level of education, and tobacco smoking. Detailed medical history of diabetes was taken from patients including age at diagnosis of DM, hypoglycemic attacks, type of treatment (insulin or oral hypoglycemic drug), history of hypertension, dyslipidemia or other diseases and medications taken. Any complaint suggestive for presence of sensory peripheral neuropathy such as tingling, burning pain or numbness in hands and feet was recorded. All studied participants were subjected to thorough clinical examination, systolic and diastolic blood pressure and anthropometric assessment (weight and height measurements).

#### Assessment of cognitive function with its domains

was carried out using Addenbrooke's Cognitive Examination III (ACE III) test: It assesses five cognitive domains: attention, memory, verbal fluency, language and visuospatial abilities [12]. It is the most reliable and validated Arabic form cognitive scale and freely available to be applied. Objective mild cognitive impairment (MCI) was considered if ACE III score was less than 88 [13]. The Trail Making Test (TMT) was also used. TMT is one of the most widely used instruments in neuropsychological assessment as an indicator of speed of cognitive processing and executive functioning. It assesses attention, concentration, psychomotor speed, cognitive shifting and complex sequencing function by measuring the time required to connect a series of sequentially numbered and lettered circles. The test consists of two parts (A and B). The direct score of each part is represented by the time of completion of the tasks. Shorter time indicates better cognitive function [14].

#### Assessment of T2DM patients' lifestyle

Patients were given a designed questionnaire to assess their lifestyle regarding nutritional habits, physical, social and mental activities and the opinion about the best way to motivate them for changing their lifestyle.

#### Baseline laboratory analysis

Five ml blood sample was withdrawn under complete aseptic technique from each participant, after fasting for at least 10 hours at the beginning of the study. It was divided into dry plain and lavender ethylene diamine tetra acetic acid (EDTA) vacutainer tubes. Level of glycated hemoglobin (HbA1c) and total blood cholesterol were assessed using Olympus-au-400 chemistry auto-analyzer. MDA concentrations in serum was determined using ELISA kit based on the competitive binding enzyme immunoassay technique according to Bird and Draper [15]. Erythrocytes were examined for the enzymatic antioxidant activity of catalase, GPx and GR [16].

### Lifestyle modification interventional program

An interventional study for health education (HE) and lifestyle modification program was carried out. The studied participants were divided into two groups: those who accepted to participate in the intervention lifestyle modification program and those who refused to participate, being the conventional group. The conventional group received their regular conventional medical follow-up. There was no significant difference between the two groups regarding age, gender, education and mean BMI, HbA1c and cognitive function. It was assumed that the conventional group after 6 months of follow-up will differ in their health behavior and subsequently in their disease prognosis compared with the interventional group. The follow-up of the participants was carried out during the period from 1 April 2018 till 31 January 2019. The program aimed at altering the unhealthy long-term habits to a healthy lifestyle. Its contents were following a healthy diet depending on glycemic index and CHO counting, monitoring the level of lipids in the blood to adjust cholesterol levels, regular physical activity for at least 30 minutes; 3–5 days per week, weight loss and maintaining an appropriate weight, monitoring and controlling the blood pressure, salt restriction, taking care of the feet, smoking cessation, practicing mental activity, and avoid stress. This program was implemented through the following:

#### Designing two health educational booklets

After identifying the T2DM participants' needs, the research team designed two HE booklets. The first booklet was titled “Diabetes mellitus and how to control its complications”. The booklet includes information about T2DM disease and how it affects different body systems leading to DM complications. It also includes information on normal and abnormal level of fasting blood glucose and glycated hemoglobin (HbA1c). It demonstrates how to control and prevent DM complications, stressing on healthy nutrition and carbohydrate counting, physical activities, strict adherence to medical regimen and avoiding stress. The booklet referred to the American Diabetes Association Guidelines 2017 and National Diabetes Committee 2015 [17,18]. The second booklet titled “Mild cognitive impairment and how to prevent” it includes information about definition of cognitive function, MCI and Alzheimer and its relation with T2DM and risk factors for cognitive impairment and how to control and improve cognitive function. The booklet referred to the Attention and working memory [19] and Noticing memory problems [20].

#### Patient education program implementation

**Figure 1.**
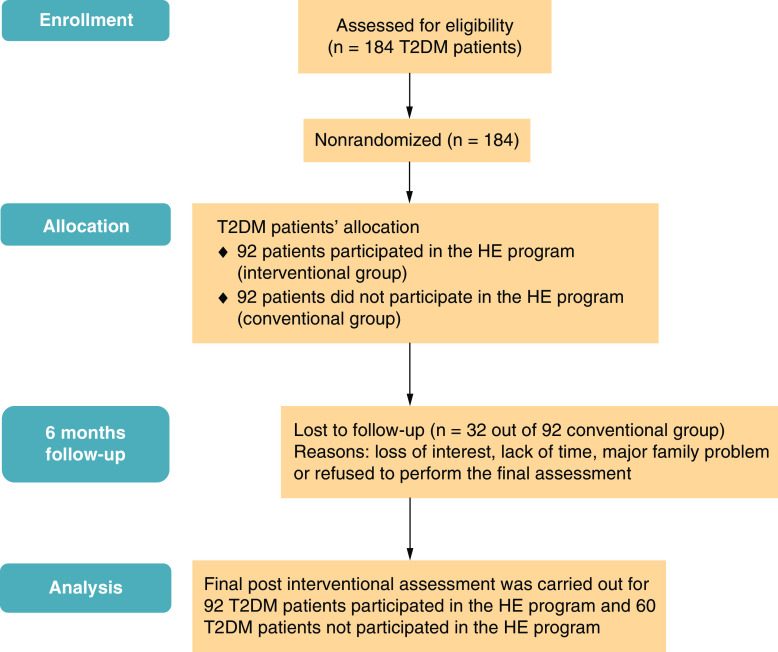
A flowchart of the study implementation. HE: Health education; T2DM: Type 2 diabetes mellitus.

The 92 participants attended the HE program session and continued the follow-up monitoring for 6 months (interventional group). The 92 T2DM patients who not attended the lifestyle modification program, received their conventional medical treatment (conventional group). Only 60/92 of them accepted to be followed up for 6 months, with dropout about 35%. Figure 1 shows a flowchart of the study implementation.

##### Face to face & group discussion health education sessions

Were carried out to encourage lifestyle modification. All the studied patients were invited to attend the HE sessions. Seven sessions were held at NRC hall over a period of nearly 2 months explaining the two designed booklets. HE lectures were presented by the public health team and a diabetic expert who shared in the group discussion to explain and answer the questions related to management of T2DM. The HE program included information about controlling DM, food important for brain health and different intellectual games to improve cognition.

##### Dissemination of educational materials

The prepared booklets were distributed to all attendants with stressing on reading them and referring to these booklets when facing any problems. It was stressed to the participants on the importance of these two booklets in improving their scientific information on DM and MCI.

##### Reassessment of laboratory analysis

Three ml of blood sample was withdrawn under complete aseptic technique from each participant, after fasting for at least 10 hours at the time of HE. It aimed to assess fasting blood glucose (FBG), HbA1c and total blood cholesterol just before the intervention and were reassessed three months during the intervention to be as an incentive for the patients to monitor the progress they attained due to lifestyle modification. Each participant was asked to receive his laboratory results personally from the research team. This allowed the discussion and the interpretation of his results and thus re-encouraging or giving him any needed advice to maintain his adherence to the lifestyle modification program.

##### Clinical follow-up & management

Were carried out by specialists in endocrinology and neurology depending on laboratory results and the patients' complaint.

##### Individualized medical nutrition therapy

Each participant was asked to fulfil a dietary form with what he/she was going to take daily for a least 3 consecutive days and to be delivered after that in order to advise and convince them to change their dietary habits through individual face-to-face interview. Every patient was taught how he could regulate his diet depending on the result of laboratory analysis and carbohydrate count.

##### Individualized biweekly follow-up scheme

Was also distributed as a guide for the targeted healthy behaviors needed to be changed and it allows self-monitoring of their achieved progress. Over a period of 6 months, the participants were followed up through mobile phone, using designed sheet, to assess patients' compliance and adherence to the lifestyle modification program.

##### WhatsApp phone application group

The participants were welcomed to contact the research team whenever they have any inquiry concerning lifestyle or treatment modifications. Through this group, 12 audio electronic messages were recorded and distributed through WhatsApp group. These messages summarized the content of the two booklets, to be an easily accessible way for the patients to help them follow the HE. Tips for healthy habits as well as exercises for mental activities were also uploaded in this group.

##### Final reassessment of clinical status & laboratory profile

A face-to-face interview was carried out with the two studied participant groups. They were 92 in the interventional group and in the conventional group, 32 out of 92 participants were lost during the follow-up as shown in the flow chart Figure 1. This was due to loss of interest, lack of time, major family problem or refused to perform the final assessment. They fulfilled a final sheet to assess their compliance to the program. To evaluate the impact of the lifestyle modification program: ACE III test, TMT with its two parts A and B tests, clinical, weight, blood pressure, HbA1c, lipid profile, oxidant, and antioxidant reassessment were carried out.

## Statistical analysis

Data entry and analysis were done using statistical program for social science (SPSS) version 23 for windows SPSS; Inc. (IL, USA). Continuous data were expressed as mean and standard deviation. Chi-square was computed for comparisons between qualitative variables and Fisher's exact test was used when appropriate. For comparing the difference between before and after follow-up, paired *t*-test was used for quantitative data and McNemar test for qualitative data. Multivariate logistic analysis was performed to identify the significant predictive risk factors for cognitive impairment and glycemic uncontrolled among the studied patients after 6 months of follow-up; p-value is statistically significant when it is <0.05 and considered statistically highly significant if it is <0.01.

## Results

In the present study, 184 T2DM patients were participated in baseline assessment. There were homogenous baseline characteristics between patients who participated in the HE program and those who did not, with no significant difference between them regarding age, gender, education, marital state, BMI, blood pressure, creatinine, lipid panel, DM duration, anti-glycemic therapy, DM treatment regularity, glycemic control, or MCI, p > 0.05 (Table 1).

**Table 1.** Baseline demographic and laboratory results among the studied T2DM patients.

Variables	Total T2DM patients (n = 184)	Interventional group (n = 92)	Conventional group (n = 92)	p-value
Age (mean ± SD)	53.8 ± 5.4	53.90 ± 4.8	53.60 ± 5.9	0.702
Age category, n (%)				
<45	12 (6.5)	5 (5.4)	7 (7.6)	
45–49.9	25 (13.6)	9 (9.8)	16 (17.4)	0.094
50–54.9	58 (31.5)	36 (39.1)	22 (23.9)	
55–59.9	64 (34.8)	33 (35.9)	31 (33.7)	
60–64.9	18 (9.8)	5 (5.4)	13 (14.1)	
>=65	7 (3.8)	4 (4.3)	3 (3.3)	
Sex				
Male	74 (40.2)	32 (34.8)	42 (45.7)	
Female	110 (59.8)	60 (65.2)	50 (54.3)	0.176
Education				
Secondary	88 (47.8)	44 (47.8)	44 (47.8)	1.000
University-post graduate	96 (52.2)	48 (52.2)	48 (52.2)	
Marital state				
Single	8 (4.3)	5 (5.4)	3 (3.3)	
Married	152 (83.6)	77 (83.7)	75 (81.5)	
Widowed	18 (9.8)	8 (8.7)	10 (10.9)	0.708
Divorced	6 (3.3)	2 (2.2)	4 (4.3)	
ACEIII	88.9 ± 5.2	89.7 ± 5.2	88.2 ± 5.1	0.054
HbA1c	7.9 ± 1.8	7.9 ± 1.7	8.2 ± 1.9	0.144
Fasting blood sugar	154.8 ± 57.3	158.59 ± 58.7	151.1 ± 55.871	0.383
Lipid panel, (mean ± SD)				
Total cholesterol (mg/dl)	204.8 ± 46.1	208.4 ± 42.6	201.0 ± 49.4	0.285
Triglycerides (mg/dl)	134.9 ± 85.5	130.5 ± 67.7	139.5 ± 100.5	0.474
HDL (mg/dl)	42.1 ± 11.9	43.5 ± 11.4	40.7 ± 12.3	0.1279
LDL (mg/dl)	134.9 ± 38.8	134.2 ± 35.7	135.5 ± 41.9	0.821
Creatinine	0.95 ± 0.22	0.95 ± 0.19	0.95 ± 0.24	0.892
BMI (kg/m^2^)	32.3 ± 5.4	32.9 ± 5.7	31.6 ± 5.2	0.105
Blood pressure, (mean ± SD)				
Systolic blood pressure (mmHg)	123.7 ± 16.3	122.9 ± 17.8	124.5 ± 14.8	0.526
Diastolic blood pressure (mmHg)	77.5 ± 10.7	76.6 ± 11.0	78.4 ± 10.3	0.207
Diabetes duration (years), mean (SD)	9.7 ± 9.3	10.3 ± 10.6	9.2 ± 7.9	0.440
Type of DM treatment				
No treatment	16 (8.7)	7 (7.6)	9 (9.8)	0.873
Oral treatment	136 (73.9)	69 (75.0)	67 (72.8)	
Insulin	32 (17.4)	16 (17.4)	16 (17.4)	
DM treatment regularity				
Yes	145 (78.8)	75 (81.5)	70 (76.1)	0.668
No	23 (12.5)	10 (10.9)	13 (14.1)	
New diagnosed	16 (8.7)	7 (7.6)	9 (9.8)	

SD: Standard deviation; T2DM: Type 2 diabetes mellitus.

Table 2 shows the behavioral and lifestyle changes after participating in the HE program among the interventional group according to gender. The percentage of modification ranged between 48.9% for decreased intake of margarine and 100.0% for increased intake of fresh vegetables. More than 80.0% of the studied patients were able to modify their lifestyle regarding increased intake of healthy snacks, drinking water, physical activities, and decreased intake of carbohydrates, fried food, sugar, sweats, and desert. Females experienced a significant increase in the intake of oats and avoided intake of yellow cheese compare to males, p < 0.01. While, males experienced a significant decrease in the intake of margarine compared with females, p < 0.01.

**Table 2.** Behavioral and lifestyle changes after participating in the lifestyle modification program among the interventional group according to gender.

Behavioral change	Total (n = 92), n (%)	Males (n = 29), n (%)	Females (n = 63), n (%)	p-value
Avoided intake of yellow cheese				0.003
Modified	61 (66.3)	13 (44.8)	48 (76.2)	
Not modified	31 (33.7)	16 (55.2)	15 (23.8)	
Increased intake of fresh vegetables				–
Modified	92 (100.0)	29 (100.0)	63 (100.0)	
Not modified	0 (0.0)	0 (0.0)	0 (0.0)	
Increased intake of oats				
Modified	61 (66.3)	13 (44.8)	48 (76.2)	0.003
Not modified	31 (33.7)	16 (55.2)	15 (23.8)	
Decreased intake of margarine				
Modified	45 (48.9)	20 (69.0)	25 (39.7)	0.009
Not modified	47 (51.1)	9 (31.0)	38 (60.3)	
Decreased intake of carbohydrate				
Modified	82 (89.1)	27 (93.1)	55 (87.3)	0.406
Not modified	10 (10.9)	2 (6.9)	8 (12.7)	
Decreased eating fried food				
Modified	87 (94.6)	28 (96.6)	59 (93.7)	0.569
Not modified	5 (5.4)	1 (3.4)	4 (6.3)	
Decreased intake of sugar intake				
Modified	76 (82.6)	27 (93.1)	49 (77.8)	0.072
Not modified	16 (17.4)	2 (6.9)	14 (22.2)	
Decreased intake of soft drinks				
Modified	65 (70.7)	24 (82.8)	41 (65.1)	0.084
Not modified	27 (29.3)	5 (17.2)	22 (34.9)	
Decreased intake of sweets and deserts				
Modified	82 (89.1)	27 (93.1)	55 (87.3)	0.406
Not modified	10 (10.9)	2 (6.9)	8 (12.7)	
Increased intake of healthy snacks				
Modified	86 (93.5)	25 (86.2)	61 (96.8)	0.055
Not modified	6 (6.5)	4 (13.8)	2 (3.2)	
Increased water intake				
Modified	86 (93.5)	26 (89.7)	60 (95.2)	0.314
Not modified	6 (6.5)	3 (10.3)	3 (4.8)	
Increased physical activities				
Modified	74 (80.4)	23 (79.3)	51 (81.0)	0.854
Not modified	18 (19.6)	6 (20.7)	12 (19.0)	
Practiced Intellectual games				
Modified	71 (77.2)	22 (75.9)	49 (77.8)	0.839
Not modified	21 (22.8)	7 (24.1)	14 (22.2)	

p-value is not-significant if >0.05, significant if <0.05 and highly significant if <0.01.

Table 3 presents the comparison between before and after 6 months follow-up among the interventional and the conventional T2DM groups. The patients participated in the program had significant improvement in the mean levels of weight, BMI, cognitive function, glycemic control, MDA, GPx, and lipid panel after 6 months of the interventional follow-up compared with the baseline, p < 0.01. There was significant improvement in the mean levels of cognitive function, SBP, glycemic control, GR, and lipid panel, after 6 months follow-up, among the interventional group compared with conventional group, p < 0.05. While, among the conventional group, there was a significant increase in the mean FBS, GR and HDL after 6 months follow-up, p < 0.05.

**Table 3.** Clinical and laboratory assessment before and after 6 months follow-up among the studied T2DM patients.

Variables	Interventional group		Conventional group			
	Baseline data n = 92 mean ± SD	After 6 months follow-up n = 92 mean ± SD	Baseline data n = 92 mean ± SD	After 6 months follow-up n = 60 mean ± SD	p-value^‡^	p-value^§^
Weight (kg)	85.9 ± 16.9	83.8 ± 16.1	84.8 ± 13.9	83.5 ± 13.3	0.792	0.543
Weight change from baseline	-2.12 ± 4.7		-0.14 ± 1.0			
p-value^†^	<0.001		0.205			
BMI	33.3 ± 6.2	32.5 ± 5.9	31.1 ± 4.9	31.2 ± 5.4	0.357	0.186
BMI change from baseline	-0.74 ± 1.95		-0.055 ± 0.27			
p-value^†^	0.001		0.142			
HbA1c	7.9 ± 1.7	6.9 ± 1.4	8.2 ± 1.9	8.3 ± 2.1	0.144	0.001
HbA1c change from baseline	-0.939 ± 1.5		-0.22 (1.2)			
p-value^†^	<0.001		0.152			
Fasting blood sugar	158.6 ± 58.7	150.9 ± 53.4	149.3 ± 53.8	166.1 ± 65.4	0.263	0.122
Fasting blood sugar change from baseline	-7.7 ± 44.1		15.8 ± 50.6			
p-value^†^	0.102		0.019			
ACEIII	89.7 ± 5.3	92.04 ± 4.4	87.3 ± 4.9	87.7 ± 5.6	0.002	<0.001
ACEIII change from baseline	2.4 ± 5.1		0.40 ± 2.7			
p-value^†^	<0.001		0.162			
Total cholesterol (mg/dl)	208.4 ± 42.6	182.4 ± 39.5	203.9 ± 54.2	211.9 ± 64.8	0.536	0.001
Cholesterol changes from baseline	-26.1 ± 33.6		6.9 ± 34.8			
p-value^†^	<0.001		0.129			
Triglycerides (mg/dl)	130.5 ± 67.7	118.4 ± 50.2	145.0 ± 91.2	151.1 ± 86.0	0.228	0.004
Triglyceride changes from baseline	-12.0 ± 55.8		4.53 ± 41.8			
p-value^†^	0.041		0.408			
High-density lipoprotein (mg/dl)	43.5 ± 11.4	51.7 ± 13.3	41.3 ± 12.9	47.2 ± 13.2	0.230	0.042
High-density lipoprotein changes from baseline	8.3 ± 14.4		5.2 ± 12.7			
p-value^†^	<0.001		0.002			
Low-density lipoprotein (mg/dl)	134.2 ± 35.7	106.1 ± 33.4	136.5 ± 42.9	131.1 ± 46.8	0.691	<0.001
Low-density lipoprotein changes from baseline	-28.1 ± 33.7		-4.82 ± 35.8			
p-value^†^	<0.001		0.304			
MDA (nmol/ml)	3.4 ± 2.8	1.0 ± 0.7	4.4 ± 4.1	4.2 ± 4.1	0.441	<0.001
Malondialdehyde change from baseline	2.4 ± 2.8		-0.2 ± 1.4			
p-value^†^	<0.001		0.469			
GR (ng/ml)	1.8 ± 1.1	2.1 ± 1.2	1.7 ± 0.9	2.0 ± 0.8	0.369	0.459
Glutathione reductase change from baseline	0.4 ± 1.4		0.3 ± 0.6			
p-value^†^	0.057		0.015			
GPx (u/ml)	9.2 ± 4.5	10.9 ± 5.2	11.7 ± 7.5	12.3 ± 6.7	<0.001	0.498
Glutathione peroxidase change from baseline	1.7 ± 5.4		0.6 ± 3.6			
p-value^†^	0.020		0.289			
SBP	122.89 ± 17.7	127.1 ± 13.8	124.3 ± 15.0	122.0 ± 12.9	0.569	0.022
SBP change from baseline	4.16 ± 20.4		-0.46 ± 2.7			
p-value^†^	0.056		0.182			
DBP	76.59 ± 11.0	78.3 ± 8.8	78.6 ± 10.3	77.4 ± 8.5	0.220	0.491
DBP change from baseline	1.74 ± 11.2		0.28 ± 3.3			
p-value^†^	0.144		0.586			

† p-value between baseline and after 6 months in each group.

‡ p-value between the two groups at baseline.

§ p-value between the two groups after 6 months.

p-value is not-significant if >0.05, significant if <0.05 and highly significant if <0.01.

Paired t test was carried out for only 60 patients in the conventional group.

SD: Standard deviation.

There were significant improvements in the mean total score of ACE III and three of its cognitive domains; fluency, language, and shape recognition, and TMT (A and B) among the interventional group compared with their baseline assessments, p < 0.01. Moreover, the interventional group experienced significant improvements in the mean total score of ACE III, all cognitive domains, and TMT (A and B) compared with conventional group, p < 0.05, Table 4.

**Table 4.** Cognitive function domains and total score before and after 6 months follow-up among the studied T2DM patients.

Variables	Interventional group		Conventional group			
	Baseline data n = 88 mean ± SD	After 6 months follow-up n = 88 mean ± SD	Baseline data n = 88 mean ± SD	After 6 months follow-up n = 60 mean ± SD	p-value^‡^	p-value^§^
Attention	17.6 ± 0.9	17.7 ± 0.8	17.0 ± 1.1	17.3 ± 1.0	<0.001	0.007
Attention changes from baseline	-0.1 ± 1.0		-0.3 ± 0.7			
p-value^†^	0.295		0.002			
Memory	23.5 ± 2.2	23.8 ± 2.5	23.0 ± 2.9	22.9 ± 2.7	0.084	0.035
Memory changes from baseline	-0.3 ± 2.8		0.1 ± 2.0			
p-value^†^	0.410		0.702			
Fluency	8.4 ± 2.2	9.3 ± 1.9	7.4 ± 2.2	7.6 ± 2.2	0.007	<0.001
Fluency changes from baseline	-0.9 ± 2.1		-0.2 ± 1.3			
p-value^†^	<0.001		0.207			
Language	24.9 ± 1.5	25.8 ± 0.8	24.8 ± 1.6	25.0 ± 1.5	0.629	<0.001
Language changes from baseline	-0.9 ± 1.4		-0.2 ± 1.1			
p-value^†^	<0.001		0.282			
Shape recognition	14.9 ± 1.3	15.5 ± 1.1	13.8 ± 1.8	14.3 ± 1.9	<0.001	<0.001
Shape recognition changes from baseline	-0.6 ± 1.6		-0.5 ± 1.1			
p-value^†^	0.001		0.001			
Total ACE III	89.2 ± 5.1	92.0 ± 4.4	86.1 ± 5.6	87.1 ± 5.8	<0.001	<0.001
Total ACE III changes from baseline	-2.8 ± 5.2		-1.1 ± 3.8			
p-value^†^	<0.001		0.033			
Trail A (time/second)	49.1 ± 16.5	45.0 ± 14.7	54.7 ± 24.7	52.0 ± 17.4	0.059	0.008
Trail A changes from baseline	4.2 ± 14.2		2.7 ± 15.6			
p-value^†^	0.009		0.191			
Trail B (time/second)	106.1 ± 42.6	94.0 ± 34.5	125.4 ± 46.2	120.1 ± 43.4	0.002	0.002
Trail B changes from baseline	12.1 ± 40.1		5.4 ± 20.1			
p-value^†^	**0.007**		0.050			

† p-value between baseline and after 6 months in both groups.

‡ p-value between the two groups at baseline.

§ p-value between the two groups after 6 months.

p-value is not-significant if >0.05, significant if <0.05 and highly significant if <0.01.

ACE III = Addenbrooke's Cognitive Examination III.

Paired t test was carried out for only 60 patients in the conventional group.

Risk factors affecting the mean level of HbA1c among the studied T2DM patients before and after 6 months follow-up are shown in Table 5. It reveals that the percent of patients with uncontrolled HbA1c significantly decreased among patients in intervention group with university education, receiving oral hypoglycemic, DM treatment regularity and those having normal cognitive function irrespective of age, gender, p < 0.01. While, there was no significant change in percent of patients with uncontrolled HbA1c among patients in the conventional group, p > 0.05.

**Table 5.** Risk factors affecting the mean level of HbA1c among the studied T2DM patients before and after 6 months follow-up.

Variables	Interventional group		Conventional group			
	Baseline data Total = 92	After 6 months follow-up Total = 92	p-value^‡^	Baseline data Total = 92	After 6 months follow-up Total = 60	p-value^§^
	HbA1c >7 n = 59 n/Total (%)	HbA1c >7 n = 31 n/Total (%)		HbA1c >7 n = 63 n/Total (%)	HbA1c >7 n = 42 n/Total (%)	
Age (years) <55 ≥55	37/50 (74.0) 22/42 (52.4)	22/50 (44.0) 9/42 (21.4)	0.001 0.004	34/44 (77.3) 29/48 (60.4)	22/27 (81.5) 20/33 (60.6)	0.125 1.000
p-value^†^	0.049	0.028		0.116	0.961	
Sex Male Female	22/29 (75.9) 37/63 (58.7)	10/29 (34.5) 21/63 (33.3)	0.002 0.002	34/47 (72.3) 29/45 (64.4)	23/31 (74.2) 19/29 (65.5)	0.687 0.500
p-value^†^	0.160	1.00		0.503	0.576	
Education Secondary University	30/44 (68.2) 29/48 (60.4)	22/44 (50.0) 9/48 (18.8)	0.096 <0.001	38/52 (73.1) 25/40 (62.5)	25/36 (69.4) 17/24 (70.8)	0.125 1.000
p-value^†^	0.516	0.002		0.366	1.00	
Type of DM treatment^¶^ Oral Insulin	45/69 (65.2) 11/16 (68.8)	21/69 (30.4) 8/16 (50.0)	<0.001 0.250	40/61 (65.0) 11/18 (65.6)	21/36 (58.3) 9/12 (75.0)	0.289 1.000
p-value^†^	1.000	0.154		0.387	0.493	
DM treatment regularity^¶^ Regular Irregular	49/75 (65.3) 7/10 (70.0)	25/75 (33.3) 4/10 (40.0)	<0.001 0.453	43/66 (65.2) 8/14 (57.1)	28/42 (66.7) 4/6 (66.7)	0.687 1.00
p-value^†^	1.000	0.729		0.559	1.000	
DM duration DM duration ≥10 years DM duration <10 years	25/38 (65.8) 34/54 (63.0)	16/38 (42.1) 15/54 (27.8)	0.022 0.001	25/37 (67.6) 38/55 (69.1)	17/21 (81.0) 25/39 (64.1)	0.500 0.031
p-value^†^	0.781	0.152		0.877	0.174	
Baseline ACE III MCI Normal cognition	19/36 (52.8) 35/50 (70.0)	15/36 (41.7) 13/50 (26.0)	0.388 <0.001	29/46 (63.0) 31/41 (75.6)	21/31 (67.7) 18/24 (75.0)	0.687 0.500
p-value^†^	0.118	0.163		0.250	0.765	

† p-value within each item in each group.

‡ p-value between baseline and after 6 months among T2DM patients in intervention group.

§ p-value between baseline and after 6 months among T2DM patients in conventional group.

¶ 165 patients were on DM treatment and the rest [19] were newly diagnosed.

p-value is not-significant if >0.05, significant if <0.05 and highly significant if <0.01.

DM: Diabetes mellitus.

Risk factors affecting the cognitive function among the studied T2DM patients before and after 6 months follow-up are shown in Table 6. It reveals that the percent of patients with MCI (ACE III ≤88) significantly decreased among patients in intervention group after 6 months follow-up with secondary education, receiving oral hypoglycemic, DM treatment regularity and those having normal cognitive function irrespective of age, gender, p < 0.01. While, there was no significant change in percent of patients with MCI among patients in the conventional group, p > 0.05.

**Table 6.** Risk factors affecting cognitive function among T2DM patients the studied T2DM patients before and after 6 months follow-up.

Variable	Interventional group			Conventional group		
	Baseline data Total = 88	After 6 months follow-up Total = 88	p-value^‡^	Baseline data Total = 88	After 6 months follow-up Total = 60	p-value^§^
	MCI (ACEIII ≤88) n = 36 n/Total (%)	MCI (ACEIII ≤88) n = 11 n/Total (%)		MCI (ACEIII ≤88) n = 46 n/Total (%)	MCI (ACEIII ≤88) n = 31 n/Total (%)	
Age (years) <55 ≥55	22/49 (44.9) 14/39 (35.9)	7/49 (14.3) 4/39 (10.3)	0.001 0.021	23/44 (52.3) 23/44 (52.3)	16/30 (53.3) 15/30 (50.0)	1.000 0.500
p-value^†^	0.394	0.570		1.000	0.796	
Sex Male female	8/29 (27.6) 28/59 (47.5)	1/29 (3.4) 10/59 (16.9)	0.039 0.001	17/47 (36.2) 29/41 (70.7)	12/33 (36.4) 19/27 (70.4)	1.000 0.727
p-value^†^	0.075	0.072		0.001	0.009	
Education Secondary University	27/40 (67.5) 9/48 (18.8)	7/40 (17.5) 4/48 (8.3)	<0.001 0.227	35/52 (67.3) 11/36 (30.6)	24/36 (66.7) 7/24 (29.2)	1.000 1.000
p-value^†^	<0.001	0.195		0.001	0.004	
Type of DM treatment^¶^ Oral Insulin	24/66 (36.4) 8/15 (53.3)	11/66 (16.7) 0/15 (0.0)	0.011 –	34/58 (58.6) 8/17 (47.1)	21/35 (60.0) 5/13 (38.5)	0.125 1.000
p-value^†^	0.225	0.089		0.474	0.183	
DM treatment regularity^¶^ Regular Irregular	30/71 (42.3) 2/10 (20.0)	11/71 (15.5) 0/10 (0.0)	0.001 –	35/62 (56.5) 8/14 (57.1)	22/42 (52.4) 4/6 (66.7)	0.125 1.000
p-value^†^	0.178	0.181		0.962	0.511	
DM duration DM duration ≥10 years DM duration <10 years	14/36 (38.9) 22/52 (42.3)	4/36 (11.1) 7/52 (13.5)	0.013 0.01	17/34 (50.0) 29/54 (53.7)	17/34 (50.0) 29/54 (53.7)	1.000 0.727
p-value^†^	0.748	0.743		0.735	0.735	
Baseline HbA1c HgAC1>7 HgAc1<=7	19/56 (33.9) 17/32 (53.1)	5/56 (8.9) 6/32 (18.8)	**0.001** **0.013**	29/60 (48.3) 17/28 (60.7)	29/60 (48.3) 17/28 (60.7)	1.000 0.500
p-value^†^	0.078	0.180		0.279	0.279	

† p-value within each item in each group.

‡ p-value between baseline and after 6 months among interventional group.

§ p-value between baseline and after 6 months among conventional group.

¶ 165 patients were on DM treatment and the rest [19] were newly diagnosed.

p-value is not-significant if >0.05, significant if <0.05 and highly significant if <0.01.

DM: Diabetes mellitus; MCI: Mild cognitive impairment.

Table 7 shows the results of logistic regression analysis for identifying the predicting variables affecting glycemic control and cognitive function after 6 months follow-up among the studied T2DM patients. Lower education, uncontrolled baseline glycemic control, DM duration >10 years, and receiving only conventional therapy were predictive factors for uncontrolled DM. As regard MCI, female gender, low baseline cognitive function (ACE III <88), and receiving only conventional therapy were the predictors for MCI, p < 0.01.

**Table 7.** Logistic regression analysis for identifying the predicting variables for uncontrolled DM and mild cognitive impairment after 6 months follow-up.

Noncomplying lifestyle modification program	B	Adjusted odds ratio (AOR)	p-value	95% CI for AOR	
				Lower	Upper
Uncontrolled DM (HbA1c >7)					
Secondary education	0.996	2.707	0.043	1.031	7.109
Baseline HbA1c >7	0.799	2.223	<0.0001	1.613	3.065
DM duration ≥10 years	1.078	2.939	0.030	1.107	7.798
Receiving only conventional therapy	1.431	4.184	0.006	1.514	11.560
Constant	-11.672	0.000	0.000		
Mild cognitive impairment (ACE III <88)					
Female gender	1.574	4.824	0.016	1.344	17.317
Baseline cognitive function (ACE III <88)	2.384	10.849	<0.0001	3.355	35.085
Receiving only conventional therapy	2.443	11.512	<0.0001	3.611	36.698
Constant	-9.618	0.000	0.000		

Variable(s) entered in each model: age, gender, education, baseline HbA1c, baseline cognitive function, DM treatment, DM duration, DM regular treatment, BMI, participation in lifestyle modification program.

p-value is not-significant if >0.05, significant if <0.05 and highly significant if <0.01.

AOR: Adjusted odds ratio; CI: Confidence interval; DM: Diabetes mellitus.

## Discussion

According to the International Diabetes Federation, Egypt is listed as number 9 in the top countries for the number of people with diabetes mellitus [1]. The present study assessed the impact of a lifestyle modification program on glycemic control and cognitive function among T2DM patients. It was detected that the prevalence of baseline uncontrolled DM among the studied participants was 66.3% with no significant difference between interventional and conventional groups. In addition to pharmacologic therapy of diabetes, it is important to include patient themselves as an important component in the treatment [21–23]. The majority of T2DM (84.7%) did not achieve the target glycemic control [11], which may be related to many factors in particularly poor lifestyle behavior and sedentary lifestyle as they are also risk factors for insulin resistance [24,25]. Most of diabetes guidelines recommended patient education and exercise as essential elements in management of diabetes and prevention of its complications [9,10]. By the end of the 6 months follow-up, the current study found that the majority of males and females in the intervention group had modified their dietary habits and practiced physical exercise during the implementation of the lifestyle modification program. The majority of participants reported positive modification for all items of healthy food in particularly increasing intake of fresh vegetables, healthy snacks, and drinking water and avoiding intake of diet rich in sugar-sweeting beverages, carbohydrates, and fried food. According to the American diabetes association, healthy lifestyle modification has a fundamental role in management of diabetes care and should include nutrition, physical exercise, and psych-social care [26] similar to the current study. Healthy dietary behavioral by increasing intake of fresh vegetables, oats and avoiding eating food rich in saturated fats and sugar were correlated with lower levels of HbA1c, triglyceride and lipid profile [26–28]. The current study stressed on the healthy eating patterns, took into consideration personal lifestyle habits, social and cultural factors. Current intervention lifestyle program showed an increase in the percent of patients who practiced physical exercise. According to the guidelines of American Diabetes Association and many randomized clinical trials and systematic review; physical activity is one of the cornerstones of diabetes management [26,28–33].

Before HE, the mean level of HbA1c among the interventional diabetic patients was 7.9 ± 1.7 compared with conventional group (8.2 ± 1.9), p > 0.05. After follow-up, there was a significant improvement in the mean level of HbA1c (6.9 ± 1.4) among the interventional group (p < 0.0001), while among the conventional group there was no significant change (8.3 ± 2.1), p > 0.05. The logistic analysis revealed that T2DM patients on conventional therapy, those had DM duration over 10 years, lower education, uncontrolled baseline glycemic control (HbA1c >7), experienced higher significant predictive risk of uncontrolled DM with AOR 4.2, 2.9, 2.7, and 2.2, respectively. It was reported from a large prospective observational study [34] that the incidence rates of the complications of T2DM were associated with glycemic control and each 1% reduction in the mean value of updated HbA1c (after adjusted for possible confounders at diagnosis of diabetes) was associated with 21% reduction of the deaths related to diabetes, 14% reduction in myocardial infarction, and 37% reduction for microvascular complications. Many studies reported similar result of improving the glycemic control among participants with T2DM using different models of lifestyle and nutritional education methods and different research methods [10,22,30,32,35] have shown significant decline in HbA1c after the intervention. Similarly, numerous studies used single group [34,36] or non-randomized two groups [36,37] concluded significant improvement in HbA1c. It is now evident that the risk of complications either macrovascular or microvascular among T2DM return to higher levels of HbA1c.

In the present study, body weight and BMI was significantly decreased after the program among interventional group. The mean level of weight and BMI among the interventional diabetic group was 85.9 ± 16.9 and 33.2 ± 6.2, respectively, before HE, with a significant improvement after follow-up (the mean level of weight and BMI were 83.8 ± 16.1 and 32.5 ± 5.9, respectively), p < 0.0001. While among the conventional group there was no significant change before and after follow-up, p > 0.05. Similar findings were reported in different countries and regions [22,35,36]. T2DM are characterized by overweight and obesity, which are modifiable risk factors for diabetic and cardiac patients because abdominal adiposity induces insulin resistance [38]. The visceral fat is diabetogenic, it causes lipotoxicity, secretes adipokines, and accumulation of macrophages that release inflammatory cytokines as IL-6 and TNF-α, which impair insulin sensitivity. Storage of excess fat in subcutaneous tissues alleviates the risk of insulin resistance and T2DM [24,25]. Therefore, almost all guidelines for diabetes management recommend weight loss for overweight and obese T2DM patients as the first step in T2DM management [38,39]. Body weight reduction improves glycemic control, lipids, and other cardiovascular risk factors [29,39].

As the majority of the studied participants modified their dietary habits, improved their physical practice with a significant decrease in their body weights, these eventually might be related to the detected improvement in their lipid profiles. There were significant declines in total cholesterol, triglycerides, and LDL among interventional group (p < 0.05), but there was no significant improvement among the conventional group, p > 0.05. This may indicate the success of the applied program specially there was no other health education programs neither on the public media, nor in the work place (NRC), and the endocrinology professor in the outpatient clinic was the same for both groups. We assumed that expected bias due to convenient sampling including selection bias and sampling bias. Among the intervention group, as expected, patients' response and interaction with the program is different. For example, every diabetic patient had his own medical condition, cultural and social norms surrounding health behaviors, low health literacy levels, incomplete perceptions of health, educational disparities, time constraints, lack of access to healthy foods and physical activity options. Similarly, it was reported in several international studies that patient education has an effective role in glycemic control and improving lipid profiles [22,24,28,36]. Sanaeinasab *et al.* in Iran [22] reported significant improvements in total cholesterol, HDL, LDL and triglycerides among the intervention diabetic patients after 3 months of the HE intervention program. On the contrary, Di Onofrio *et al.* in Italy [37] reported insignificant differences in lipid profile among the intervention group after 9 months of the nutrition intervention program. Moreover, numerous researches reported significant difference only in total cholesterol and triglycerides [40,41]. Other researches showed only significant improvement in total cholesterol [30] or in triglycerides [31,42]. These variations in the effect of lifestyle intervention programs may return to many factors as duration and component of the intervention programs, age of the participants, extent of adherence of participants to adopt the intervention, degree of weight loss, using lipid lowering drugs, levels of blood lipid profile before the starting the intervention program.

Oxidative stress has been linked to the pathogenesis of many human chronic conditions e.g., diabetes, hypertension and atherosclerosis [43]. Uncontrolled DM patients are considered to be under oxidative stress [44]. The increased oxidative stress in diabetes is responsible for insulin resistance [45] and for development of diabetic complications as microvascular and cardiovascular complications [43,44,46,47]. The current study revealed significant decline in the mean value of MDA (oxidant enzyme) and significant increase in the mean value of GPx (antioxidant enzyme) among the interventional group, p < 0.05, but there was no significant improvement among the conventional group, p > 0.05. It has been documented that implementing lifestyle and dietary weight loss program with or without exercise for obese T2DM patients have positive effect on oxidative stress [48]. In line with our results, He *et al.* [49] studied the effect of individualized dietary intervention program on oxidative stress and found a significant reduction in MDA levels [50]. Similarly, Abd El-Kader and Al-Dahr [51] investigated the effect of weight loss program on the oxidative markers and reported significant decline in MDA and significant increase in GPx among the intervention group.

It is documented that uncontrolled blood glucose levels among DM patients leads to many cerebrovascular and neuropathic complications (declined level of IGF, impaired vascular reactivity, and reduced cerebral blood flow) which affect brain function. Therefore, these patients are more prone to cognitive decline and exhibits worse cognitive ability with evidence of structural and functions abnormalities on brain imaging [4,5,52–57]. These complications are linked to insulin resistance, excessive glycation and oxidation causing inflammation and apoptosis in neurons, increased platelet aggregation, endothelial dysfunction, as well as impaired fibrinolysis and eventually increase the risk of dementia and stroke. Diabetes additionally decreases the energy and nutrients important for producing cellular proteins, lipids, and nucleic acids leading to cell starvation and death [58–62]. There was a significant improvement of the cognitive function domains (fluency, language, and shape recognition), total score of ACEIII, and TMT A and B among the interventional group, p < 0.01. While, there was mild significant improvement among the conventional group only in the mean total ACE III score, p < 0.05. The logistic analysis indicated that T2DM patients on conventional therapy, those with baseline mild cognitive impairment, and female gender experienced higher significant predictive risk of cognitive impairment after 6 months follow-up with AOR 11.5, 10.8 and 4.8, respectively. There was no a hierarchy between investigators and study participants as the participants were working in different administration departments or staff members from different NRC institutes. Types of works of the participants: professors, engineers, dentists, accountants, technicians, nurses, lawyers, clerks, directors, managers, secretary and security officials. Previous studies reported that there is inconsistency about the efficacy of physical exercise or dietary patterns intervention and improvement in cognitive functions [63,64]. Some studies reported a significant association between lifestyle intervention and improvement of cognitive functions [65–69]. On the contrary, other studies found no association between lifestyle intervention (physical or dietary or both) and cognitive benefit [70,71]. Proper controlling of hyperglycemia among diabetic patients and targeting risk factors in particularly cardiovascular risk factors, glycemic control and lifestyle modification (healthy diet and physical activity) might reduce the risk of MCI among T2DM. The successful outcomes in the current study could be attributed to the willing of the studied participants in intervention group to change their lifestyle. In addition, the program was implemented through different HE methodology including face-to-face interview and group discussion cessions. The investigators designed and distributed two health educational booklets on DM and cognitive function. Over a period of 6 months, the participants were followed up clinically and were contacting through mobile phone-to assess their compliance and adherence to the program and were received HE audio messages and posts through WhatsApp. Finally frequent laboratory reassessment as an incentive for the patients to monitor the progress they attained due to their lifestyle modification.

## Conclusion

Implementing a lifestyle modification intervention program, using hybrid methods of HE as face to face, group discussion, printing materials, electronic messages, and mobile WhatsApp, application was successful. The program contents include maintaining healthy diet depending on glycemic index and CHO counting, adjusting cholesterol level, regular physical activity for at least 30 minutes; 3–5 days per week, weight loss and maintaining an appropriate weight, controlling the blood pressure, smoking cessation and practicing mental activity. After 6 months, there was a significant improvement in glycemic control, cognitive function, oxidant and antioxidant and lipid profile levels among patients participating in the program but not among those remained on the conventional therapy. Further studies on a large scale or a community basis are recommended.

## Limitations of the study

The sampling for the current study was a convenient sampling (non-probability sampling method). Studied patients were allocated to the interventional group (T2DM patients wishing to participate in the lifestyle modification program) and a conventional group who did not participate in the program (control group). So, we assumed that the expected bias would be of such as the selection and sampling bias. A convenience sample doesn't provide a representative result. The study was carried on NRC employees, so the results cannot be generalized.

**Summary points**

- This study aimed at assessing the impact of lifestyle modifications on glycemic control and cognitive function of Type II diabetes mellitus (T2DM) patients.
- A lifestyle modification program was implemented on a sample of T2DM patients. The participants were followed up after 6 months.
- Significant improvements were observed in the intervention group in the mean levels of HbA1c, oxidant and antioxidant, lipid profile, BMI, total score of ACE III, and Trail (A and B).
- T2DM patients on conventional therapy, those who had DM duration over 10 years, lower education, and uncontrolled baseline glycemic control, experienced higher significant predictive risk of persistent uncontrolled DM.
- T2DM patients on conventional therapy, those with baseline mild cognitive impairment, and female gender experienced higher significant risk of cognitive impairment.
- In conclusion, lifestyle modification is an important process for improving T2DM glycemic control and in improving the cognitive function among the diabetic patients.
- Further studies on a large scale or a community basis are recommended.
